# Domestic

**Published:** 1841-05-15

**Authors:** 


### DOMESTIC.

*Death of Dr. Miner, of Connecticut.*—The eastern papers announce the death of Thomas Miner, M. D., of Middletown, Connecticut, in the 64th year of his age—a highly esteemed and learned physician, and connected with the Medical Department of Yale College, and many of the benevolent institutions of the state.

---

Errata.—The following omissions occurred in the column for rain in the Meteorological Register for April:—

.328 on the 10th.

.010 on the 24th.

---

### HEALTH OF THE CITY.

Interments *in the City and Liberties of Philadelphia, from the* 1st *to the* 8th *of May*, 1841.

| Diseases. | Adults. | Children. |
|---|---|---|
| Abscess of throat, | 0 | 1 |
| Apoplexy, | 1 | 0 |
| Cancer of womb, | 1 | 0 |
| Croup, | 0 | 2 |
| Congestion of lungs, | 1 | 0 |
| Congestion of brain, | 1 | 0 |
| Cholera morbus, | 1 | 0 |
| Consumption of the lungs, | 19 | 4 |
| Convulsions, | 0 | 3 |
| Dropsy, | 2 | 0 |
| —— abdominal, | 0 | 1 |
| —— head, | 0 | 4 |
| —— breast, | 2 | 1 |
| Disease of brain, | 0 | 2 |
| —— heart, | 0 | 1 |
| —— lungs, | 0 | 1 |
| —— medulla oblongata, | 0 | 1 |
| —— bronchia, | 0 | 1 |
| Dysentery, | 1 | 0 |
| Debility, | 0 | 1 |
| Enlargement of the heart, | 0 | 1 |
| Fever, | 0 | 1 |
| —— bilious remittent, | 0 | 1 |
| Gangrene, | 0 | 1 |
| Inflammation of the brain, | 1 | 5 |
| —— bronchi, | 1 | 5 |
| —— lungs, | 4 | 4 |
| —— stomach, | 1 | 1 |
| —— stomach and bowels, | 1 | 0 |
| —— liver, | 0 | 1 |
| —— bladder, | 1 | 0 |
| *Carried forward*, | 39 | 46 |

| Diseases. | Adults. | Children. |
|---|---|---|
| *Brought forward*, | 39 | 46 |
| Inflammation of peritonæum, | 1 | 0 |
| Inanition, | 0 | 1 |
| Intemperance, | 1 | 0 |
| Marasmus, | 1 | 3 |
| Malformation, | 0 | 2 |
| Measles, | 0 | 9 |
| Mania, | 1 | 0 |
| Neglect, | 0 | 1 |
| Old age, | 2 | 0 |
| Ossif. of heart, | 1 | 0 |
| Palsy, | 3 | 0 |
| Pleurisy, | 1 | 1 |
| Rickets, | 0 | 1 |
| Scrofula, | 1 | 1 |
| Spitting of blood, | 1 | 0 |
| Small pox, | 0 | 7 |
| Still-born, | 0 | 11 |
| Ulcerated sore throat, | 0 | 1 |
| Unknown, | 3 | 0 |
| Total, 139— | 55 | 84 |

| Of the above, there were | |
|---|---|
| under 1 year | 38 |
| From 1 to 2 | 13 |
| 2 to 5 | 12 |
| 5 to 10 | 11 |
| 10 to 15 | 3 |
| 15 to 20 | 6 |
| 20 to 30 | 11 |
| 30 to 40 | 15 |
| 40 to 50 | 1 |
| 50 to 60 | 7 |
| 60 to 70 | 6 |
| 70 to 80 | 4 |
| 80 to 90 | 2 |
| Total, | 139 |

Of the above there were 9 from the almshouse, and 28 people of colour, which are included in the total amount.

